# A typical case of neurofibromatosis

**DOI:** 10.11604/pamj.2022.42.171.35492

**Published:** 2022-07-01

**Authors:** Mayuri Amol Deshpande, Amol Madhav Deshpande

**Affiliations:** 1 Department of Kayachikitsa, Mahatma Gandhi Ayurved Medical College and Research Centre, Datta Meghe Institute of Medical Sciences (Deemed to be University) Salod (H), Wardha, Maharashtra, India; 2 Department of Rachana Sharir, Mahatma Gandhi Ayurved Medical College and Research Centre, Datta Meghe institute of Medical Sciences (Deemed to be University), Salod (H), Wardha

**Keywords:** Neurofibromatosis, neurofibromatosis type 1, Recklinghausan’s disease

## Image in medicine

Neurofibromatosis type 1 is also known as Recklinghausan’s disease. It is an autosomal dominant disorder. The mutation causes large number of nodules all over the body. A patient of 42 years old age came to the Outpatient Department (OPD) of Mahatma Gandhi Ayurved college hospital and research centre, Salod (H), Wardha, Nagpur, Maharashtra, India with tingling and numbness over palm and foot. The patient has taken lots of medicine for the neurofibromas. But the medicinal trials were gone in vain. So he abandonned the therapeutics for the neurofibromas. He said that he had these lumps from the age of 8 years. Neurofibromatosis with numerous flesh coloured neurofibromas on chest and abdomen (A). Neurofibromatosis with numerous flesh coloured neurofibromas on back (B). Neurofibromatosis with numerous flesh coloured neurofibromas on the anterior aspect of forearms (C). Neurofibromatosis with numerous flesh coloured neurofibromas on the posterior aspect of forearms (D).

**Figure 1:**
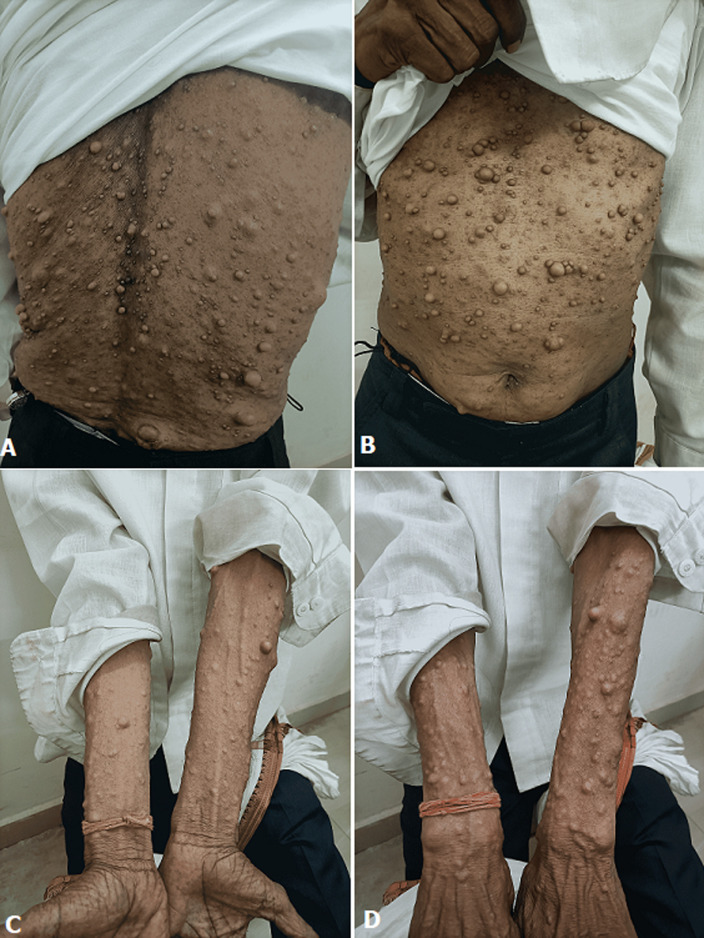
neurofibromatosis with numerous flesh coloured neurofibromas on: (A) back; (B) abdomen and chest; (C) the anterior aspect of forearms; (D) the posterior aspect of forearms

